# Phytotoxic and Genotoxic Effects of Copper Nanoparticles in Coriander (*Coriandrum sativum*—Apiaceae)

**DOI:** 10.3390/plants8010019

**Published:** 2019-01-14

**Authors:** Alya O. AlQuraidi, Kareem A. Mosa, Kalidoss Ramamoorthy

**Affiliations:** 1 Department of Applied Biology, College of Sciences, University of Sharjah, Sharjah P.O. Box 27272, UAE; u16101516@sharjah.ac.ae (A.O.A.); kramamoorthy@sharjah.ac.ae (K.R.); 2 Department of Biotechnology, Faculty of Agriculture, Al-Azhar University, Cairo, Egypt

**Keywords:** *Coriandrum sativum*, phytotoxicity, genotixicity, CuNP, RAPD, XRF

## Abstract

Engineered metal nanoparticles have been widely used in several applications that may lead to increased exposure to the environment. In this study, we assessed the phytotoxic effect of various concentrations of copper nanoparticles CuNP, (200, 400 and 800 mg/L) on coriander (*Coriandrum sativum*) plants grown hydroponically. *C. sativum* plants treated with CuNP demonstrated decreased biomass and root length in comparison to control untreated plants. Additionally, decreased levels of photosynthetic pigments (chlorophyll a and b) were also seen in *C. sativum* plants treated with CuNP, as well as damage to the *C. sativum* root plasma membrane as demonstrated by Evan’s blue dye and increased electrolyte leakage. Moreover, our results exhibited increased levels of H_2_O_2_ and MDA on *C. Sativum* plants treated with CuNP. X-Ray Fluorescence (XRF) analysis confirmed that *C. sativum* treated with CuNP accumulated the latter in plant root tissues. Random amplified polymorphic DNA (RAPD) analysis confirmed the genotoxic effect of CuNP, which altered the *C. sativum* genome. This was shown by the different banding pattern of RAPD. Overall, our results exhibited that CuNP is toxic to *C. sativum* plants.

## 1. Introduction

Nanoparticles are nano-scale particles or atomic aggregates with at least one dimension between 1 and 100 nm. Nanoparticles have been a great source of concern in recent years due to their extensive usage in many industries (including agriculture). They have been used in many applications in agriculture including pesticides, fungicides and fertilizers [1]. Depending on their origin, further distinctions can be made. There are naturally occurring, incidental and engineered nanoparticles (ENPs). Major naturally occurring nanoparticles can be found in the atmosphere: volcanic eruptions, desert surfaces, dust from cosmic sources located in the solar system such as iron oxides that can be found in water, soil and sediments of earth crust [2]. Incidental nanoparticles are produced as side products of anthropogenic activities whereas Engineered Nanoparticles (ENPs) are produced intentionally for their extensive usage in various implications including biomedical, electronic and industrial. They express specific sizes and shapes, which can enter the environment through air, water and soil [3].

Due to the wide range of implications, the utilization of ENPs in various fields (including industrial, medical, mechanical and agriculture) can result in increased exposure to our environment [4]. Examples of widely used ENPs include silver nanoparticles (AgNP), which are present in products like cosmetics, clothing and household items [5]. Additionally, iron nanoparticles (FeNP) are found in concrete additives [6]. Lead nanoparticles (PbNP) are found in automotive exhaust converters [6], while cobalt nanoparticles (CoNP) are used in biomedical and health science applications [6,7,8]. Copper nanoparticles (CuNP) are involved in the production of temperature and pressure sensors [7,8] Regardless of their benefits to several industries, questions on how ENPs are affecting the environment and interacting with living organisms have been put forward in recent times. Due to their extensive utilization, nanoparticle traces are inadvertently released at different stages of their production and usage, raising concerns of their potential risks. Although several ENPs have exhibited beneficial effects on crops, phytotoxic assessment would still be recommended depending on the size, shape, zeta potentials, concentrations, transportation, transformation, dosage and the plant species of the involved nanoparticles. Recently, several studies and experiments have been performed to evaluate the effect of ENPs on the environment in terms of direct exposure and long-term accumulation. The size of the ENPs can determine the amount of toxicity, with a larger surface area allowing increased interaction with the environment [9].

Several metals act as micronutrients including copper are essential in small quantities for the proper functioning of biological systems. In plants, copper is particularly important for the synthesis of lignin and several other enzyme systems such as Cu/Zn superoxide dismutase (SOD), cytochrome c oxidase, amino oxidase, laccase, plastocyanin and polyphenol oxidase [10], photosynthesis and the metabolism of carbohydrates and proteins [11]. However, high concentrations of such metals can adversely lead to reduced growth, altered metabolism, less biomass and metal accumulation [12]. Furthermore, if metals are present in the form of nanoparticles, the resulting effects would be largely unpredictable due to size and shape differences [13]. As plants are an important part of the environment, the potential negative effects of metal nanoparticles in their case have been an increasing concern. In the past decade, several studies have described the phytotoxic effects of ENPs on plants [14]. However, divulging detailed mechanisms of phytotoxicity have still been elusive. Therefore, toxicological studies should be undertaken to evaluate the fate of nanoparticles, their toxic effect, transformation and distribution in plants, in addition to their effect on the physiological, biochemical and molecular aspects.

As copper is as an essential constituent in plants, CuNPs have been utilized as antimicrobial agents for biocidal activity [15,16]. In addition, CuNPs are used in agriculture as an effective nano-metallic fungicide application in crops to protect against fungal diseases [17,18].

*Curcubita pepo* (zucchini) treated with CuNP and bulk copper powder showed 77% and 64% of root length reduction, respectively, when compared to control plants. Additionally, their exposure also resulted in a 90% reduction in biomass [19]. CuNPs have also been observed to significantly reduce seedling growth in *Elsholtzia splendens* grown in a hydroponic system treated with different concentrations of copper particles; 100, 200, 500, 1000 mg/L CuONP, 1000 mg/L CuOBPs, and 0.5 mg/L soluble Cu [20], as well as reducing root and shoot growth, and chlorophyll and carotenoids contents of Indian mustard [21]. Other than morphological changes, CuNPs can also affect the biochemical content of plants as demonstrated by reduced chlorophyll content and increased hydrogen peroxide and lipid peroxidation resulting in increased reactive oxygen species (ROS) production in cucumbers [22]. The effect of the copper nanoparticle also extends to the DNA level by inducing DNA damage in radish and grasses [23], as well as cucumbers [22].

Coriander *(Coriandrum sativum)* is an annual herb and food leaf crop, which is used in many parts of the world for food and medicinal purposes, and therefore was chosen for this study to evaluate the phytotoxic and genotoxic effect of CuNP. *C. sativum* was treated with CuNPs of size ~20 nm with different concentrations (200, 400 & 800 mg/L) and the plants were grown in a hydroponic system to maintain the dosage of the nanoparticle. Hence, the impact of CuNP was analyzed at morphological, physiological, and molecular levels.

## 2. Results

### 2.1. CuNP Decreased C. sativum Root Length, Biomass, and Chlorophyll Content

**Figure 1:**
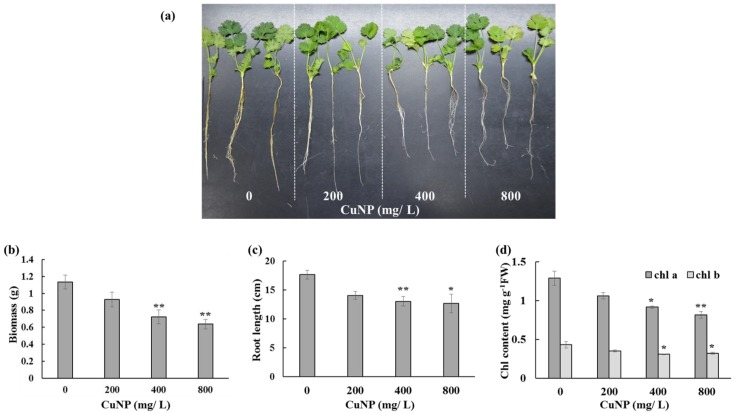
Phenotypic and chlorophyll analysis of *C. sativum* plants after 7 days of exposure to 200, 400 and 800 mg/L of CuNPs. (**a**) Morphology of roots and shoots of *C. sativum* control and treated plants. (**b**) Biomass of *C. sativum* plants. (**c**) Root length of *C. sativum* plants. (**d**) Chlorophyll a and b contents of *C. sativum* plants. The values are mean ± se (*n* = 3). Statistically significant difference was calculated at * *p* ≤ 0.05, ** *p* ≤ 0.01.

Plants treated with different concentrations of CuNPs exhibited differences in the biomass and root length after seven days of the treatment (Figure 1a,b). Treated roots exhibited a different morphology with weaker, thinner and reduced adventitious roots, whereas the shoots showed no changes. There was no significance difference between control and 200 mg/L CuNP treated plants. However, 400 and 800 mg/L of CuNP treated plants showed statistically significant reduction with more than 35% biomass decrease compared with the control untreated plants (Figure 1b). Additionally, root length analysis between control plants showed enhanced root growth and the production of more adventitious roots, whereas treated (200, 400 and 800 mg/L) plants had reduced root growth. Differences between control and treated plants with different CuNPs concentrations were dose dependent, the reduction being statistically significant with around 17% root length decrease in 400 mg/L and 800 mg/L CuNPs treated plants (Figure 1c).

Treated plants showed a decrease in both chlorophyll a and chlorophyll b when compared to control plants, but this response varied with the concentration of CuNP. At the lowest levels of CuNP (200 mg/L), the decrease was not significant. However, plants treated with 400 mg/L and 800 mg/L of CuNP exhibited significant decrease in both chlorophyll a and b contents (Figure 1d).

### 2.2. CuNP Caused Membrane Damage on C. sativum Plants

**Figure 2:**
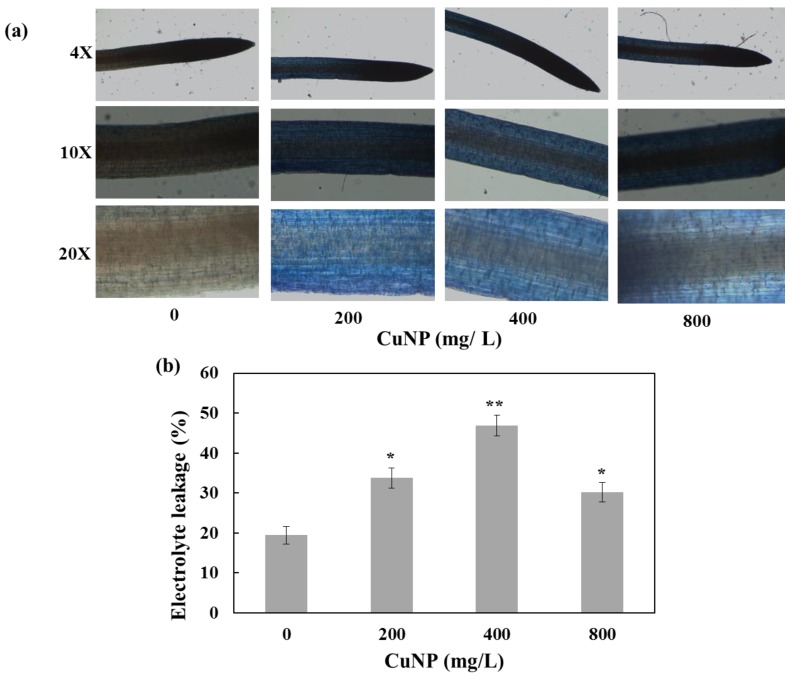
Evan’s blue and electrolyte leakage analysis of *C. sativum* plants after 7 days of exposure to 200 mg/L, 400 mg/L and 800 mg/L of CuNPs. (**a**) plasma membrane integrity of roots monitored under 4×, 10× and 20× magnifications using Evan’s blue staining. (**b**) electrolyte leakage analysis of *C. sativum* plants. The values are mean ± se (*n* = 3). Statistically significant difference was calculated at * *p* ≤ 0.05, ** *p* ≤ 0.01.

Root samples of the control and treated *C. sativum* were observed under light microscopy after Evan’s blue staining. The control plants were observed to be normal under microscopy and therefore did not take the stain. In contrast, treated plants were stained according to the concentration of CuNP (Figure 2a), which demonstrated that the membrane had been damaged. Electrolyte leakage analysis showed an increase in the conductivity of the leakage solution of the 200 mg/L, 400 mg/L and 800 mg/L treated roots by 34%, 46% and 30%, respectively (Figure 2b). This indicated the damage at the level of *C. sativum* root plasma membranes due to CuNP treatment. 800 mg/L CuNP treated plants showed reduced electrolyte leakage compared to 200 mg/L and 400 mg/L CuNP treated plants, possibly due to higher toxic levels of CuNP efflux from the roots.

### 2.3. CuNP Increased Hydrogen Peroxide and Malondialdehyde Content in C. sativum Plants

**Figure 3:**
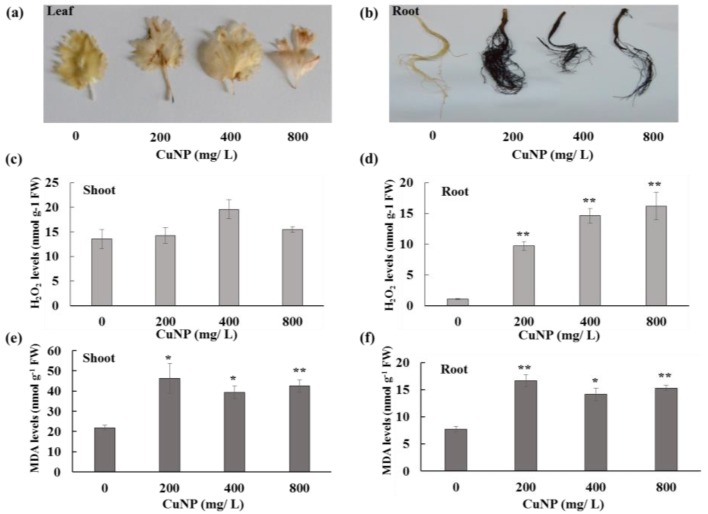
H_2_O_2_ and MDA content analysis of *C. sativum* plants after 7 days of exposure to 200, 400 and 800 mg/L of CuNPs. (**a**) DAB staining as a preliminary test for detection of H_2_O_2_ in shoots and (**b**) roots. (**c**) H_2_O_2_ levels in shoots and (**d**) roots. (**e**) MDA levels in shoots and (**f**) roots. The values are mean ± se (*n* = 3). Statistically significant difference was calculated at * *p* ≤ 0.05, ** *p* ≤ 0.01.

Shoots and roots were stained with 3, 3′- diaminobenzidine (DAB) to observe H_2_O_2_ production due to different concentrations of CuNP. Treated plants displayed brown spots on the leaves and brownish black roots, whereas control plants exhibited no changes in both shoots and roots (Figure 3a,b). Furthermore, hydrogen peroxide (H_2_O_2_) content was measured in both shoots and roots of *C. sativum* control and CuNP treated plants. Figure 3c,d shows that there were no significant differences in H_2_O_2_ levels between control and treated shoots. However, all the CuNP treated roots had significantly higher amounts of H_2_O_2_ compared to control untreated root tissues (approximately 10 to 16 -fold increase).

Regarding Malondialdehyde (MDA), in this case, CuNP treated plant shoot and root tissues had approximately 2-fold higher MDA levels in comparison to control plants (Figure 3e,f).

### 2.4. C. sativum Plants Accumulated more CuNP in Root Tissues

**Figure 4:**
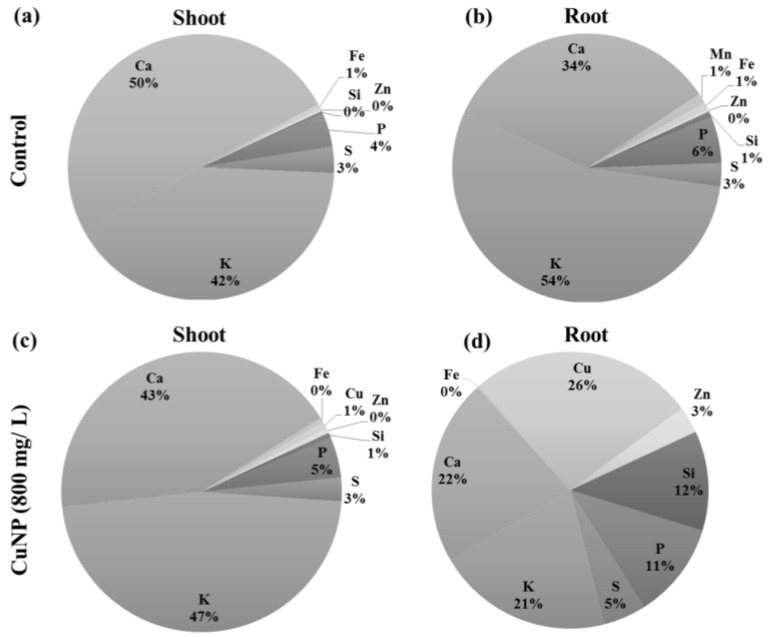
Elemental analysis of *C. sativum* plants after 7 days of exposure to 800 mg/L of CuNPs by XRF. (**a**) control shoot (**b**) control root (**c**) 800mg/L CuNPs treated shoot and (**d**) 800 mg/L CuNPs treated roots. Elemental profiles were obtained from three biological replicates.

Other than copper, elements of the Hoagland solution, such as Cl, Ca, Si, K, S, and P, were also detected (Figure 4). Remarkably, more copper was detected in root tissues compared to shoot tissues as demonstrated by XRF analysis (Figure 4c,d). The elemental profile of shoot and root tissues can vary in control tissues. Due to CuNP treatment, the profiles were changed in both shoot and root tissues of *C. sativum* plants in comparison to control plants.

### 2.5. CuNP Induced Genotoxicity in C. sativum Plants

**Figure 5:**
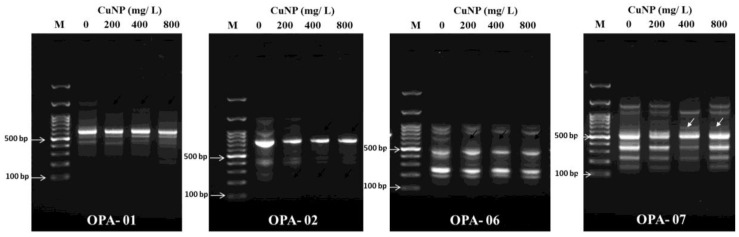
Random amplified polymorphic DNA (RAPD) analysis of *C. sativum* plants after seven days of exposure to 200 mg/L, 400 mg/L and 800 mg/L of CuNPs. All DNA of control and treated plants were amplified with OPA-1, OPA-2, OPA-6 and OPA-7. The white arrows indicate the disappearance and appearance of bands between control and treated plants.

Random Amplified Polymorphic DNA (RAPD) was performed to assess CuNP phytotoxicity effect at the genomic level of *C. sativum* (Figure 5). Extracted genomic DNA from the roots of both control and CuNP treated samples were amplified using several sets of primers (OPA-01, OPA-02, OPA-06 and OPA-07). The amplified control sample of the OPA- 01 primer showed seven bands ranging between 200 to 1200 bps. For the 200 mg/L, 400 mg/L and 800 mg/L CuNP treatments, the amplified DNA demonstrated the same bands as the control DNA samples, except that all treatments had lost one band at 1200 bps. Control sample DNA for the OPA-02 primer amplified several bands ranging between 200 to 1200 bps. However, samples treated with 400 and 800 mg/L CuNP exhibited absence of the band at 1000 bps. Control sample DNA amplified with the OPA-06 primer presented eight bands ranging between 150 bps to 1000 bps. Samples treated with 200 mg/L, 400 mg/L and 800 mg/L CuNP showed one missing band at 700 bps. In addition, control sample DNA amplified with the OPA-07 primer presented six bands ranging between 150 bps to 1200 bps. Samples treated with 200 mg/L demonstrated the same pattern as controls whereas 400 mg/L and 800 mg/L CuNP presented an additional band at 700 bps. Overall, the RAPD results demonstrated that CuNPs made significant changes in the genome of the *C. sativum* plants.

## 3. Discussion

*C. sativum* grown hydroponically allows more accessibility to the plant and easier controlled manipulation of nutrients when compared to soil due to complex ion interactions [24]. Coriander plants treated with different concentrations of CuNP (200, 400 and 800 mg/L) for seven days exhibited a decrease in root length when compared to non-treated plants. The root length was significantly reduced at all concentrations. In our earlier report, we studied the effect of CuNPs on cucumber (*C. savius*) plants in which phytotoxic effects of CuNPs were demonstrated with concentrations of 50 mg/L, 100 mg/L and 150 mg/L CuNPs. Interestingly, our preliminary experiments with coriander did not show any phytotoxic effects utilizing the same concentrations of CuNPs (data not shown) even though the same CuNPs were utilized in the earlier cucumber plants study. When we increased the concentration to the reported concentration here (200 mg/L, 400 mg/L and 800 mg/L), clear phototoxic effects were seen. These variations are clearly due to the effect of the plant species itself. Hence, coriander plants were able to tolerate CuNP levels without producing the phytotoxic effects observed in cucumber plants. Moreover, coriander’s ability to tolerate and remediate toxic metals has been reported recently. Adsorption capacity and efficiency of coriander in removal of Pb^2+^, Cd^2+^ ions and turbidity from simulated contaminated water was reported [25]. Additionally, *C. sativum* plants showed considerable potential for phytoremediation of Pb and As from contaminated soil and water [26].

CuNP treated coriander showed brown color as well as weaker or no growth of lateral and hair roots in comparison to controls, possibly due to root accumulation of CuNP as indicated by XRF results. It was shown that rice treated with copper oxide nanoparticles exhibited reduced root and shoot length [27]. Similar results have also been reported for lettuce and alfalfa [28]. In addition, another study showed that zucchini root length was affected by CuNP [19]. This was similar to *A. thaliana*, where the plant growth and biomass was reduced when treated with different concentrations (2 mg/L, 5 mg/L, 10 mg/L, 20 mg/L, 50 mg/L, and 100 mg/L) of copper oxide nanoparticles [29].

The presence of high concentrations of endogenous H_2_O_2_ implies a very high rate of production under CuNP-induced stress. High accumulation of endogenous H_2_O_2_ levels was also evident in our CuNP stressed coriander leaves and roots, which implies that anti-oxidant enzymes are not sufficient to scavenge the excess H_2_O_2_. Similarly, this was also reported in rice and Syrian barley treated with copper oxide nanoparticle (CuONP) [30,31]. Additionally, H_2_O_2_ may act as a secondary messenger to trigger many physiological processes, as recorded under CuNP stress. Coriander plants showed high sensitivity towards CuNP and increased electrolyte leakage under CuNP treatments, the latter leading to alterations in the membrane permeability. Evan’s blue was used as a marker to demonstrate that increases in CuNP concentration augmented cell membrane damage through enhanced accumulation of the dye, resulting in cell death. This was reported in cucumber treated with both cerium oxide (CeO_2_) and lanthanum oxide (La_2_O_3_) nanoparticles [32]. This was also reflected in the MDA results, since MDA formation is related to lipid peroxidation indicating highly significant membrane damage to our treated plant cells as compared to control plant cells. Similar results were shown in Syrian barley treated with CuONP, as well as lettuce plants treated with ceria nanoparticles [31,33]. Additionally, toxic effects of CuNP are probably exerted through free radical generation, which results in the enhanced production of MDA. Metals are known to inhibit the precursor of chlorophyll called protochlorophyllide by inhibiting the enzyme protochlorophyllide reductase [34]. The reduction is mainly due to the high redox potential of metals including copper [35]. Additionally, copper is one of the essential micronutrients for plants and should be tightly regulated. Either copper deficiency or excess copper leads to variation in plant growth and development by adverse physiological processes [10]. The chlorophyll contents were measured as a photosynthetic performance, which showed a significant reduction in the 400 mg/L and 800 mg/L treatments in comparison to control plants. This might be due to damage in chloroplast membranes as a result of excess lipid peroxidation under higher oxidative stress, or changes in leaf thickness and anatomy, or as a result of reduction in the availability of mineral elements leading to depletion of Iron (Fe) as a result of antagonism between Cu and Fe uptake. This has been already shown in Brassica juncea L and Arabidopsis treated with Cu and CuNP [21,36,37].

XRF microscopy was used for elemental analysis in plant tissues semi-quantitatively (mass %). This was performed to show the elemental composition of *C. sativum* shoots and roots treated with CuNP. Micro-XRF analysis has also been used widely in earlier studies for elemental analysis, as shown when detecting CeO_2_ in the soybean epidermis [38]. In another report, micro-XRF was used to study how the CeO_2_ nanoparticles changed the allocation of calcium in kernel [39]. Moreover, it was used to map titanium oxide (TiO_2_) distribution in wheat where it accumulated in root tissues and distributed through whole plant tissues [40]. Our elemental analysis of CuNP treated samples showed 26% and 1% of copper in roots and shoots, respectively, whereas it was undetectable in control untreated plants. Interestingly, a clear increase in Si (12%) was detected in CuNP treated roots compared to 1% in control untreated roots, which may be part of the plant coping mechanism against CuNP stress as reported previously [41].

RAPD technique is used to detect polymorphisms in the genome without prior knowledge of the DNA sequence [32]. RAPD was used to assess the genotoxicity of TiO_2_ in Cucurbita pepo which demonstrated DNA changes in treated versus control conditions [42]. Moreover, it has been used to assess the genotoxicity of cerium oxide (CeO2) and titanium Oxide (TiO_2_) in Hordeum vulgare L [43]. Furthermore, the genotoxic effect of zinc oxide (ZnO) and copper oxide nanoparticle (CuO NP) has been shown in buckwheat (*Fagopyrum esculentum*) as assessed by RAPD [44]. ZnO and CeO_2_ also showed genotoxic effects in soybean plants demonstrating different DNA patterns when compared to controls [45]. Additionally, genotoxicity was demonstrated via RAPD in cucumber plants treated with CuNP [22]. In our investigation, four standard primers (OPA1, OPA4, OPA6 and OPA7) were utilized. The genomic changes were demonstrated on agarose gels as different bands that disappeared or appeared when comparing control and treated plant DNA. Appearance of new patterns may be explained by changes in the genomic DNA template stability due to mutations, large deletions or homologous recombination. Additionally, it can also indicate a change in priming sites leading to new annealing events [44]. In observing the appearance and disappearance of bands, more of the latter phenomenon was detected in our study when comparing control and treated plants. As increases in oxidative stress can lead to DNA damage, the higher presence of ROS in treated samples may cause modifications in the observed RAPD pattern [43].

In general, CuNP induced alteration of physiological, biochemical and molecular activities in *C. sativum* plants. Uptake and accumulation of CuNP caused oxidative stress that limited successful plant growth and development, which was more observable in roots than shoots due to direct contact between the membrane root and CuNPs. Additionally, CuNP induced lipid peroxidation led to membrane damage as observed by enhanced MDA concentrations.

## 4. Materials and Methods

### 4.1. Plant Materials and Nanoparticle Treatment

Coriander seeds used in this study were bought from a local market. CuNPs were purchased from Hengqiu Graphene Technology (Suzhou) Co., Ltd., Shanghai, China with a purity of 99.9% and an average particle size of 20 nm. These CuNP were characterized in our earlier study [22]. Coriander seeds (*Coriandrum sativum*) were sterilized using 10% bleach solutions for 5 min and then washed with distilled water three times. Fitted size filter papers were inserted at the bottom of sterilized petri dishes and the sterilized seeds were dispersed along 10 mL of distilled water for 10 days. Seedlings were then transferred into hydroponic media containing 20% Hoagland’s No.2 basal salt mixture (Sigma-Alrich, H2395) and allowed to acclimatize for another 2 weeks in a growth chamber maintained at temperatures of 21 °C/18 °C for 20h light/4h dark cycles, respectively. Control samples were grown in 20% Hoagland’s solution whereas treated samples were grown in various concentrations of CuNP (200, 400 and 800 mg/L) and prepared in 20% Hoagland’s No. 2 basal salt mixture by 30 min sonication for uniform CuNP dispersal. These solutions were then used to treat the plants for 7 days. Experiments were conducted using three replicates of 15 plants each. We developed a hydroponic system that contained 3 replicas.

### 4.2. Measurement of Biomass, Root Length, and Chlorophyll Contents

After 7 days of CuNP treatment, plant roots were washed carefully with distilled water and blotted in tissue paper. Plant fresh weight and root length were measured for the control and treated groups. For chlorophyll a and b analysis, 100 mg of leaf tissues were ground using ice cold mortars and pestles, incubated with 5 mL of 80% acetone for 15 min at room temperature in the dark. Samples were then dissolved in 1:1 ratio with 80% acetone and absorbance was measured by spectrophotometer (Jenway 3600, Keison products, Chelmsford, UK) at 663 nm and 645 nm for chlorophyll a and b respectively [46].

### 4.3. Root Membrane Integrity

100 mg of plant root was excised and added to 10 mL of deionized water in test tubes. The electrical conductivity was measured by an electrical conductivity meter (EC meter) after 48 h of incubation in deionized water (C_n_), and after boiling and allowing them to cool down (C_f_). Electrolyte leakage was calculated using the following formula; E_T_ % = (C_n_/C_f_) × 100 [47]. Experiments were conducted with three replicates, with each replica comprising a total of 15 plant roots. Plant root cell death was evaluated following treatment with CuNP by performing Evan’s blue staining [48]. The control and treated plant roots were stained with 0.25% *v*/*v* of Evan’s Blue in 0.1 M calcium chloride (CaCl_2_) solution for 15 min. Subsequently, roots were washed well in distilled water to remove the excess unbound dye. Root tips were analyzed under bright field microscope (Optika B-1000 BF, Ponteranica, Italy) under different magnifications.

### 4.4. Lipid per Oxidation and H2O2 Determination

Concentration of malondialdehyde (MDA) was measured for lipid peroxidation determination according to Zhou and Leul [49]. 0.1 g of roots and shoots were mixed with 0.1% (*v*/*w*) Trichloroacetic acid (TCA). The tubes were centrifuged at 12,000 *g* for 15 min at 4 °C. 800 µL of the supernatant were mixed with 2 mL of 0.5% 2-Thiobarbituric acid (TBA) diluted in 20% TCA. This mixture was incubated in a water bath maintained at 80 °C for 1 h and allowed to cool down. Tubes were centrifuged for 5 min at 12,000 *g* and 4 °C. Optical density was measured by spectrophotometer (Jenway 3600, Keison products, Chelmsford, UK) at 532 nm and 600 nm. The concentration of MDA was calculated by using an extinction coefficient of 155 mM^−1^ cm^−1^.

H**_2_**O**_2_** contents were measured as described previously [50]. 100 mg of frozen shoots and roots were homogenized with 2 mL of 0.1% TCA solution and then centrifuged at 12,000 *g* for 15 min at 4 °C. The clear supernatant (0.5 mL) was mixed with 0.5 mL of 10 mM potassium phosphate buffer (pH 7) and 1 mL of 1 M potassium iodide and optical density were measured by spectrophotometer (Jenway 3600, Keison products, UK) at 390 nm. The H_2_O_2_ content was determined using an extinction coefficient of 0.28 µm cm^−1^ and expressed as µmol·g^−1^ FW. H_2_O_2_ accumulation was detected using a 3′,3′-diaminobenzidine (DAB) assay [51]. The control and treated plant leaf and roots were stained with 1 mg·mL^−1^ DAB solution and incubated in a shaker for 4 h by covering it with foil. The tissues were washed with ethanol, acetic acid and glycerol (3:1:1) and incubated in a 90 °C water bath to bleach the chlorophyll content. Subsequently, the leaf and roots were photographed with a white background.

### 4.5. X-ray Fluorescence (XRF) Analysis of CuNP in Plant Tissues

Dry samples were prepared by drying the roots and shoots in an oven (65 °C). Using Horiba’s XGT 7200, X-ray Analytical Microscope (XAM), elemental composition of the control and CuNP treated plant samples were measured semi-quantitatively as described previously [22]. This microscope facilitates collecting spectra either from a specific spot or particular area in our samples. Oven dried control and CuNP treated plant samples were used for measuring elemental composition using XAM.

### 4.6. DNA Extraction and RAPD Analysis

DNA was extracted from root samples stored at −80 °C using a Norgen Biotek DNA extraction kit for plant/fungi according to manufacturer instructions. DNA samples were quantified using nanodrop 2000 (Thermo Fischer Scientific, Waltham, MA, USA). A similar concentration of DNA was used with Norgen Biotek Corp master mix using the RAPD primers OPA-1 (CAGGCCCTTC), OPA-2 (TGCCGAGCTG), OPA-6 (GGTCCCTGAC), and OPA-7 (GAAACGGGTG). The RAPD PCR program was set to initial denaturation at 95 °C for 3 min, followed by a 35-cycle annealing step set at 48 °C for 1 min, extension of products at 72 °C for 2 min and the final extension set at 72 °C for 7 min using the Techne PCR machine (TC-5000) (GMI Inc., Ramsey, MN, USA). PCR amplified samples were run on 2% agarose gel electrophoresis along with molecular marker (1 kb promega molecular weight marker) and photographed using the Biorad gel doc system.

## 5. Conclusions

In conclusion, to our knowledge, this is the first report focused on evaluating the phytotoxic effect of CuNP in *C. sativum*. XRF analysis demonstrated that CuNP (20 nm) had a toxic effect on *C. sativum* root length and biomass where both decreased significantly in accordance with increasing CuNP concentration, along with a significant decrease in total chlorophyll content. Moreover, damage in the membranes of treated samples was demonstrated by an increase in electrolyte leakage as observed by Evan’s blue studies. In addition, an increase in endogenous H_2_O_2_ and MDA formation was observed in *C. Sativum* plants treated with CuNP.

Furthermore, from the molecular perspective, DNA damage was assessed by utilizing the RAPD technique which showed different patterns of bands between control and treated plants. Further studies are needed to better understand the molecular mechanisms underlying CuNP transport and accumulation in important leafy herbal plants such as *C. sativum*.

## References

[1] Khot L.R., Sankaran S., Maja J.M., Ehsani R., Schuster E.W. (2012). Applications of nanomaterials in agricultural production and crop protection: A review. Crop Prot..

[2] Guo H., Barnard A.S. (2013). Naturally occurring iron oxide nanoparticles: Morphology, surface chemistry and environmental stability. J. Mater. Chem. A.

[3] Kumar P., Kumar A., Lead J.R. (2012). Nanoparticles in the Indian Environment: Known, Unknowns and Awareness. Environ. Sci. Technol..

[4] Bhatt I., Tripathi B.N. (2011). Interaction of engineered nanoparticles with various components of the environment and possible strategies for their risk assessment. Chemosphere.

[5] Fabrega J., Luoma S.N., Tyler C.R., Galloway T.S., Lead J.R. (2011). Silver nanoparticles: Behaviour and effects in the aquatic environment. Environ. Int..

[6] Bystrzejewska-Piotrowska G., Golimowski J., Urban P.L. (2009). Nanoparticles: Their potential toxicity, waste and environmental management. Waste Manag..

[7] Athanassiou E.K., Grass R.N., Stark W.J. (2006). Large-scale production of carbon-coated copper nanoparticles for sensor applications. Nanotechnology.

[8] Lee Y., Choi J.R., Lee K.J., Stott N.E., Kim D. (2008). Large-scale synthesis of copper nanoparticles by chemically controlled reduction for applications of inkjet-printed electronics. Nanotechnology.

[9] Oberdürster G. (2000). Toxicology of ultrafine particles: In vivo studies. Philos. Trans. R. Soc. Lond. Ser. A.

[10] Yruela I. (2005). Copper in plants. Braz. J. Plant Physiol..

[11] Sommer A.L. (1931). Copper as an essential for plant growth. Plant Physiol..

[12] Nagajyoti P.C., Lee K.D., Sreekanth T.V.M. (2010). Heavy metals, occurrence and toxicity for plants: A review. Environ. Chem. Lett..

[13] Tripathi D.K., Singh S., Singh S., Pandey R., Singh V.P., Sharma N.C., Prasad S.M., Dubey N.K., Chauhan D.K. (2017). An overview on manufactured nanoparticles in plants: Uptake, translocation, accumulation and phytotoxicity. Plant Physiol. Biochem..

[14] Faisal M., Saquib Q., Alatar A.A., Al-Khedhairy A.A. (2018). Phytotoxicity of Nanoparticles.

[15] Ravishankar Rai V., Jamuna Bai A., Méndez-Vilas A. (2011). Nanoparticles and Their Potential Application as Antimicrobials.

[16] Palza H. (2015). Antimicrobial Polymers with Metal Nanoparticles. Int. J. Mol. Sci..

[17] Kiaune L., Singhasemanon N. (2011). Pesticidal copper (I) oxide: Environmental fate and aquatic toxicity. Rev. Environ. Contam. Toxicol..

[18] Ponmurugan P., Manjukarunambika K., Elango V., Gnanamangai B.M. (2016). Antifungal activity of biosynthesised copper nanoparticles evaluated against red root-rot disease in tea plants. J. Exp. Nanosci..

[19] Stampoulis D., Sinha S.K., White J.C. (2009). Assay-Dependent Phytotoxicity of Nanoparticles to Plants. Environ. Sci. Technol..

[20] Shi J., Peng C., Yang Y., Yang J., Zhang H., Yuan X., Chen Y., Hu T. (2014). Phytotoxicity and accumulation of copper oxide nanoparticles to the Cu-tolerant plant Elsholtzia splendens. Nanotoxicology.

[21] Nair P.M.G., Chung I.M. (2015). Study on the correlation between copper oxide nanoparticles induced growth suppression and enhanced lignification in Indian mustard (*Brassica juncea* L.). Ecotoxicol. Environ. Saf..

[22] Mosa K.A., El-Naggar M., Ramamoorthy K., Alawadhi H., Elnaggar A., Wartanian S., Ibrahim E., Hani H. (2018). Copper Nanoparticles Induced Genotoxicity, Oxidative Stress, and Changes in Superoxide Dismutase (SOD) Gene Expression in Cucumber (*Cucumis sativus*) Plants. Front. Plant Sci..

[23] Atha D.H., Wang H., Petersen E.J., Cleveland D., Holbrook R.D., Jaruga P., Dizdaroglu M., Xing B., Nelson B.C. (2012). Copper Oxide Nanoparticle Mediated DNA Damage in Terrestrial Plant Models. Environ. Sci. Technol..

[24] Conn S.J., Hocking B., Dayod M., Xu B., Athman A., Henderson S., Aukett L., Conn V., Shearer M.K., Fuentes S. (2013). Protocol: Optimising hydroponic growth systems for nutritional and physiological analysis of Arabidopsis thaliana and other plants. Plant Methods.

[25] Kivuti N.M. (2017). Using Cilantro Leaves and Stems to Remove Lead, Cadmium and Turbidity from Contaminated Water.

[26] Gaur N., Kukreja A., Yadav M., Tiwari A. (2017). Assessment of phytoremediation ability of Coriander sativum for soil and water co-contaminated with lead and arsenic: A small-scale study. 3 Biotech.

[27] Da Costa M.V.J., Sharma P.K. (2016). Effect of copper oxide nanoparticles on growth, morphology, photosynthesis, and antioxidant response in Oryza sativa. Photosynthetica.

[28] Hong J., Rico C.M., Zhao L., Adeleye A.S., Keller A.A., Peralta-Videa J.R., Gardea-Torresdey J.L. (2015). Toxic effects of copper-based nanoparticles or compounds to lettuce (*Lactuca sativa*) and alfalfa (*Medicago sativa*). Environ. Sci. Process. Impacts.

[29] Nair P.M.G., Chung I.M. (2014). Impact of copper oxide nanoparticles exposure on Arabidopsis thaliana growth, root system development, root lignificaion, and molecular level changes. Environ. Sci. Pollut. Res..

[30] Shaw A.K., Hossain Z. (2013). Impact of nano-CuO stress on rice (*Oryza sativa* L.) seedlings. Chemosphere.

[31] Shaw A.K., Ghosh S., Kalaji H.M., Bosa K., Brestic M., Zivcak M., Hossain Z. (2014). Nano-CuO stress induced modulation of antioxidative defense and photosynthetic performance of Syrian barley (*Hordeum vulgare* L.). Environ. Exp. Bot..

[32] Dingle T.C., MacCannell D.R., Sails A., Tang Y.-W. (2015). Chapter 9—Molecular Strain Typing and Characterisation of Toxigenic Clostridium difficile. Methods in Microbiology.

[33] Gui X., Zhang Z., Liu S., Ma Y., Zhang P., He X., Li Y., Zhang J., Li H., Rui Y. (2015). Fate and Phytotoxicity of CeO_2_ Nanoparticles on Lettuce Cultured in the Potting Soil Environment. PLoS ONE.

[34] De Filippis L.F., Ziegler H. (1993). Effect of Sublethal Concentrations of Zinc, Cadmium and Mercury on the Photosynthetic Carbon Reduction Cycle of Euglena. J. Plant Physiol..

[35] Chandra R., Kang H. (2016). Mixed heavy metal stress on photosynthesis, transpiration rate, and chlorophyll content in poplar hybrids. For. Sci. Technol..

[36] Lequeux H., Hermans C., Lutts S., Verbruggen N. (2010). Response to copper excess in Arabidopsis thaliana: Impact on the root system architecture, hormone distribution, lignin accumulation and mineral profile. Plant Physiol. Biochem..

[37] Feigl G., Kumar D., Lehotai N., Tugyi N., Molnar A., Ordog A., Szepesi A., Gemes K., Laskay G., Erdei L. (2013). Physiological and morphological responses of the root system of Indian mustard (*Brassica juncea* L. Czern.) and rapeseed (*Brassica napus* L.) to copper stress. Ecotoxicol. Environ. Saf..

[38] Hernandez-Viezcas J.A., Castillo-Michel H., Andrews J.C., Cotte M., Rico C., Peralta-Videa J.R., Ge Y., Priester J.H., Holden P.A., Gardea-Torresdey J.L. (2013). In Situ Synchrotron X-ray Fluorescence Mapping and Speciation of CeO_2_ and ZnO Nanoparticles in Soil Cultivated Soybean (Glycine max). ACS Nano.

[39] Zhao L., Sun Y., Hernandez-Viezcas J.A., Hong J., Majumdar S., Niu G., Duarte-Gardea M., Peralta-Videa J.R., Gardea-Torresdey J.L. (2015). Monitoring the environmental effects of CeO_2_ and ZnO nanoparticles through the life cycle of corn (*Zea mays*) plants and in situ mu-XRF mapping of nutrients in kernels. Environ. Sci. Technol..

[40] Larue C., Laurette J., Herlin-Boime N., Khodja H., Fayard B., Flank A.-M., Brisset F., Carriere M. (2012). Accumulation, translocation and impact of TiO_2_ nanoparticles in wheat (*Triticum aestivum* spp.): Influence of diameter and crystal phase. Sci. Total Environ..

[41] Li J., Leisner S.M., Frantz J. (2008). Alleviation of copper toxicity in Arabidopsis thaliana by silicon addition to hydroponic solutions. J. Am. Soc. Hort. Sci..

[42] Moreno-Olivas F., Gant V.U., Johnson K.L., Peralta-Videa J.R., Gardea-Torresdey J.L. (2014). Random amplified polymorphic DNA reveals that TiO_2_ nanoparticles are genotoxic to Cucurbita pepo. J. Zhejiang Univ. Sci. A.

[43] Mattiello A., Filippi A., Pošćić F., Musetti R., Salvatici M.C., Giordano C., Vischi M., Bertolini A., Marchiol L. (2015). Evidence of Phytotoxicity and Genotoxicity in Hordeum vulgare L. Exposed to CeO_2_ and TiO_2_ Nanoparticles. Front. Plant Sci..

[44] Lee S., Chung H., Kim S., Lee I. (2013). The Genotoxic Effect of ZnO and CuO Nanoparticles on Early Growth of Buckwheat, Fagopyrum Esculentum. Water Air Soil Pollut..

[45] López-Moreno M.L., de la Rosa G., Hernández-Viezcas J.Á., Castillo-Michel H., Botez C.E., Peralta-Videa J.R., Gardea-Torresdey J.L. (2010). Evidence of the Differential Biotransformation and Genotoxicity of ZnO and CeO_2_ Nanoparticles on Soybean (*Glycine max*) Plants. Environ. Sci. Technol..

[46] Lichtenthaler H.K., Wellburn A.R. (1983). Determinations of total carotenoids and chlorophylls a and b of leaf extracts in different solvents. Biochem. Soc. Trans..

[47] Liu X., Huang B. (2002). Cytokinin Effects on Creeping Bentgrass Response to Heat Stress. Crop Sci..

[48] Zanardo D.I.L., Lima R.B., Ferrarese M.d.L.L., Bubna G.A., Ferrarese-Filho O. (2009). Soybean root growth inhibition and lignification induced by p-coumaric acid. Environ. Exp. Bot..

[49] Zhou W., Leul M. (1998). Uniconazole-induced alleviation of freezing injury in relation to changes in hormonal balance, enzyme activities and lipid peroxidation in winter rape. Plant Growth Regul..

[50] Velikova V., Yordanov I., Edreva A. (2000). Oxidative stress and some antioxidant systems in acid rain-treated bean plants: Protective role of exogenous polyamines. Plant Sci..

[51] Thordal-Christensen H., Zhang Z., Wei Y., Collinge D.B. (2002). Subcellular localization of H_2_O_2_ in plants. H_2_O_2_ accumulation in papillae and hypersensitive response during the barley—Powdery mildew interaction. Plant J..

